# Comparison of Modified Sarnat Staging and Thompson Score in Neonatal Hypoxic-Ischemic Encephalopathy

**DOI:** 10.7759/cureus.104350

**Published:** 2026-02-27

**Authors:** Himanshu Gohatre, Rajesh K Kulkarni, Deepali Ambike, Sandhya Haribhakta, Suryakant Mundlod, Seema Soni, Vinay Patil

**Affiliations:** 1 Pediatrics, Postgraduate Institute, Yashwantrao Chavan Memorial Hospital, Pune, IND

**Keywords:** hypoxic-ischemic encephalopathy (hie), modified sarnat staging, neonatal encephalopathy (ne), sarnat staging of neonatal encephalopathy, thompson score

## Abstract

Background

Neonatal hypoxic-ischemic encephalopathy (HIE) is a major cause of neonatal mortality and long-term neurodevelopmental morbidity. Accurate bedside neurological assessment is critical for severity stratification and timely clinical decision-making, particularly during the early postnatal period when encephalopathy evolves dynamically. This study aimed to evaluate the correlation, agreement, and time-efficiency between the Modified Sarnat Staging (MSS) system and the Thompson Score (TS) in neonates with HIE, to determine the institutional burden of HIE, and to assess experience-related efficiency characteristics associated with repeated application of both scoring systems.

Methodology

A cross-sectional observational study was conducted over an 18-month period in the neonatal intensive care unit (NICU) of Pimpri Chinchwad Municipal Corporation Postgraduate Institute & Yashwantrao Chavan Memorial Hospital, Pimpri, Pune, India. A total of 55 neonates with gestational age ≥35 weeks were enrolled based on the American College of Obstetricians and Gynecologists diagnostic criteria for HIE. The MSS system and the TS were performed at 1, 3, 6, 9, 12, and 24 hours of life, and again at discharge. The time required to complete each assessment was recorded. Severity distribution, correlation (Spearman’s rho), agreement (unweighted and quadratic-weighted kappa), heatmap visualization, and experience-related efficiency trends were analyzed using an available-case methodology.

Results

The prevalence of HIE among neonates admitted to the NICU during the 18-month study period was 3.26% (227 of 6,965 deliveries). MSS classified 9.09% of neonates as mild, 56.36% as moderate, and 34.55% as severe HIE, whereas the TS identified 40% as mild, 18.18% as moderate, and 41.81% as severe cases. Correlation between the two systems was strong across all timepoints (Spearman’s rho = 0.76-0.91). Agreement improved progressively, with quadratic-weighted kappa increasing from 0.647 at 1 hour to 0.789 at 12 hours and 0.877 at discharge. Heatmap analyses demonstrated early discordance primarily involving infants classified as mild by the TS but moderate by MSS, while severe classifications showed high concordance from the outset. The TS was consistently faster by approximately 55 seconds at all timepoints (Wilcoxon p < 10⁻⁹). Both scoring systems demonstrated marked experience-related improvements (rho = approximately -0.98 to -0.999).

Conclusions

The MSS system and the TS demonstrate strong correlation and progressively improving agreement in the assessment of neonatal HIE. The TS offers a consistent time-efficiency advantage, while early discordance at the mild-moderate boundary underscores the importance of serial neurological assessments and complementary use of both scoring systems during early clinical decision-making.

## Introduction

Neonatal hypoxic-ischemic encephalopathy (HIE) remains a major cause of neonatal mortality and long-term neurodevelopmental impairment worldwide [1-3]. It results from a significant interruption of cerebral blood flow and oxygen delivery occurring antenatally, intrapartum, or in the immediate postnatal period. Recognized risk factors include maternal hypotension or hypoxia, placental abruption, uterine rupture, prolonged or obstructed labor, cord prolapse, fetal bradycardia, meconium-stained amniotic fluid, and the need for extensive neonatal resuscitation at birth. The incidence of HIE in term and near-term infants is estimated at 1-3 per 1,000 live births, with a disproportionately higher burden in low- and middle-income countries where access to intrapartum monitoring and timely neonatal resuscitation may be limited. Survivors of HIE are at increased risk of cerebral palsy, epilepsy, cognitive impairment, and behavioral disorders, underscoring the importance of early recognition and accurate severity stratification [4].

The neuropathology of HIE follows a well-described sequence of injury. The initial hypoxic-ischemic insult results in primary energy failure due to impaired cerebral perfusion and oxygen deprivation. This is followed by a transient latent phase during which partial metabolic recovery may occur. Subsequently, secondary energy failure develops, characterized by excitotoxicity, oxidative stress, mitochondrial dysfunction, apoptosis, and neuroinflammation [5-7]. The severity and progression of brain injury are influenced not only by the magnitude and duration of the initial insult but also by antenatal factors such as placental insufficiency, intrapartum events including prolonged labor or cord compression, and postnatal conditions such as hypoxemia, metabolic disturbances, hypotension, and sepsis. As these processes evolve over hours to days, neurological signs may fluctuate, making clinical assessment inherently time-sensitive and potentially variable during the early postnatal period [8].

Accurate bedside neurological assessment, therefore, remains central to the severity staging and management of HIE, particularly because timely intervention improves survival and neurodevelopmental outcomes in infants with moderate-to-severe encephalopathy [9-11]. In this context, standardized clinical scoring systems play a critical role in grading disease severity, guiding treatment decisions, and facilitating communication among clinicians. The Modified Sarnat Staging (MSS) system classifies HIE into mild, moderate, and severe stages based on a comprehensive clinical assessment of consciousness, spontaneous activity, muscle tone, primitive reflexes, autonomic function, and seizure activity [12]. It provides a global clinical staging framework that relies on structured neurological examination and clinical synthesis. In contrast, the Thompson Score (TS) uses a 22-point numerical scoring system across nine neurological and autonomic domains, allowing quantification of encephalopathy severity and facilitating serial trend analysis [13,14]. While MSS emphasizes categorical staging based on overall clinical patterns, TS provides a more granular numerical assessment, enabling objective monitoring of evolving neurological changes over time. Structured neurological assessment, therefore, remains central to contemporary neonatal encephalopathy evaluation [15].

Despite the widespread use of both tools, important clinical uncertainties persist. Few studies have systematically compared the correlation and agreement between MSS and TS across multiple serial timepoints within the critical first 24 hours of life, when therapeutic decisions are most time-sensitive. Furthermore, limited data exist on how agreement between these systems evolves as encephalopathy stabilizes, where discordance most commonly occurs, and whether operational factors such as time required for assessment and examiner experience-related efficiency influence their practical utility in busy neonatal intensive care units (NICUs) [14,16].

Therefore, the present study was undertaken to comprehensively evaluate the relationship between the MSS and TS in neonates with HIE through serial assessments during the first 24 hours of life and at discharge. Specifically, we aimed to evaluate the correlation and agreement between the two scoring systems across serial timepoints, analyze patterns of categorical concordance and discordance, determine the institutional burden of HIE, and compare the time-efficiency and experience-related characteristics associated with repeated application of each tool. Through this integrated analysis, we sought to provide clinically relevant evidence to inform optimal use of neurological scoring systems in real-world neonatal practice.

## Materials and methods

Study design and setting

This prospective observational (non-interventional) study was conducted in the NICU of Pimpri Chinchwad Municipal Corporation Postgraduate Institute & Yashwantrao Chavan Memorial Hospital, Pimpri, Pune, India, a tertiary-care teaching hospital. The study was conducted over an 18-month period after obtaining approval from the Institutional Ethics Committee (approval number: IECPGI-030/2022). Written informed consent was obtained from parents or legal guardians before enrolment. Although serial neurological assessments were performed, analyses at each timepoint were conducted independently, consistent with a repeated cross-sectional analytical framework.

Study population

Neonates with a gestational age of ≥35 weeks who fulfilled the American College of Obstetricians and Gynecologists diagnostic criteria for HIE were eligible for inclusion. Only intramural (inborn) neonates were enrolled in the study. Neonates with major congenital anomalies, central nervous system malformations, chromosomal disorders or syndromic features, or missing essential neurological examination data were excluded.

Sample size

The minimum required sample size was calculated as 35 neonates (power 85%, α = 0.05) to detect a moderate-to-strong correlation between scoring systems. To improve the precision of correlation and agreement estimates, all eligible neonates meeting the inclusion criteria during the 18-month study period were consecutively recruited, resulting in a final sample of 55 neonates.

Neurological assessment protocol and measurement of assessment time

Serial neurological assessments were conducted at predefined postnatal time points, specifically at 1, 3, 6, 9, 12, and 24 hours of life, and again at discharge. TS severity categories were defined a priori using commonly cited thresholds, with scores of ≤10 classified as mild, 11-14 as moderate, and ≥15 as severe HIE [13,14]. MSS was performed using the National Institute of Child Health and Human Development-adapted Sarnat criteria with a structured bedside proforma [12]. All examinations were conducted by a single trained pediatrician to ensure internal consistency. The time required to complete each assessment was recorded using a standardized stopwatch, and the order of scoring was alternated to reduce sequence bias. Therapeutic hypothermia was not available at the study center during the study period.

Outcome measures

The primary outcome of the study was the correlation between the MSS and TS at each assessment time point. Secondary outcomes included agreement between the two scoring systems using unweighted and weighted kappa statistics, time required to complete each assessment, experience-related efficiency trends with repeated scoring, and the prevalence of HIE among neonates admitted to the NICU.

Handling of missing data

Missing data were primarily attributable to early discharge or death and were considered Missing Not at Random. No imputation was performed.

Statistical analysis

Data were compiled in Microsoft Excel and analyzed using Python and R statistical software executed on the Google Colaboratory platform (Google LLC, Mountain View, CA, USA). Python was provided by the Python Software Foundation (Wilmington, DE, USA), and R was provided by the R Foundation for Statistical Computing (Vienna, Austria). Statistical analyses were performed using base functions and standard statistical libraries available within these environments. Continuous variables were summarized as mean ± standard deviation or median with interquartile range, as appropriate, while categorical variables were expressed as frequencies and percentages. Spearman’s rank correlation coefficient (ρ) was used to assess the association between the ordinal MSS and continuous TS at each time point. Agreement between categorical severity classifications was evaluated using unweighted Cohen’s kappa (κ) and quadratic-weighted kappa (κw), interpreted according to Landis and Koch criteria [17]. Because assessment times were non-normally distributed, comparisons between Sarnat and Thompson assessment durations were performed using the Wilcoxon signed-rank test. Progressive efficiency gains were analyzed by correlating assessment time with patient sequence order. A two-sided p-value <0.05 was considered statistically significant.

## Results

Study population and institutional burden of hypoxic-ischemic encephalopathy

A total of 55 neonates born at ≥35 weeks’ gestation were included in the analytic cohort. During the study period, there were 6,965 live births at the institution, of which 227 neonates were initially admitted to the NICU with HIE, yielding an institutional NICU admission prevalence of 3.26%. Although neonates with mild encephalopathy were later shifted to the postnatal ward after stabilization, all cases were captured at the time of NICU admission. This represents admission prevalence rather than population-level incidence.

Among the study cohort, 58.18% were male and 41.82% were female. Male neonates were disproportionately represented in the moderate and severe HIE categories across both scoring systems. Regarding mode of delivery, 56.36% were delivered vaginally and 43.64% by emergency cesarean section, with severe HIE more frequently observed among emergency cesarean deliveries.

Severity distribution according to Modified Sarnat and Thompson classifications

As shown in Table 1, MSS classified the majority of neonates as having moderate HIE (56.36%), whereas TS identified a substantially larger proportion of neonates as mild (40.00%) and a slightly higher proportion as severe (41.81%). The distribution of severity categories, therefore, differed between the two systems, reflecting differences in category thresholds and the relative weighting of neurological signs used by each scoring method.

**Table 1:** Distribution of hypoxic-ischemic encephalopathy severity according to Modified Sarnat Staging and Thompson Score (n = 55).

Severity	Modified Sarnat, n (%)	Thompson Score, n (%)
Mild	5 (9.09%)	22 (40.00%)
Moderate	31 (56.36%)	10 (18.18%)
Severe	19 (34.55%)	23 (41.81%)
Total	55 (100%)	55 (100%)

Correlation and agreement between Modified Sarnat and Thompson scores

As shown in Table 2, MSS and TS demonstrated a strong positive correlation in severity assessment at all assessed timepoints, with Spearman’s ρ values ranging from 0.762 to 0.908 (all p < 0.0001). Correlation of encephalopathy severity classification was evident from the first hour of life and increased over time, reaching its highest value at discharge.

**Table 2:** Correlation and agreement between Modified Sarnat and Thompson classifications across timepoints.

Timepoint	n	Spearman’s ρ	95% CI	P-value	κ (unweighted)	κw (weighted)	Interpretation
1 hour	55	0.835	0.739-0.892	<0.0001	0.402	0.647	Substantial
3 hours	53	0.762	0.529-0.900	<0.0001	0.400	0.591	Moderate-substantial
6 hours	53	0.830	0.631-0.928	<0.0001	0.499	0.668	Substantial
9 hours	53	0.823	0.667-0.907	<0.0001	0.500	0.683	Substantial
12 hours	53	0.810	0.676-0.899	<0.0001	0.611	0.789	Substantial-almost perfect
24 hours	52	0.787	0.650-0.887	<0.0001	0.579	0.743	Substantial
Discharge	55	0.908	0.770-0.993	<0.0001	0.817	0.877	Almost perfect

Agreement between categorical severity classifications also improved progressively across timepoints. Quadratic-weighted kappa (κw) values increased from 0.647 at 1 hour to 0.789 at 12 hours and 0.877 at discharge, corresponding to substantial to almost perfect agreement. Unweighted kappa values showed a similar upward trend. This temporal pattern indicates increasing concordance between the two scoring systems as postnatal neurological assessments progressed.

Heatmap interpretation across timepoints

**Figure 1:**
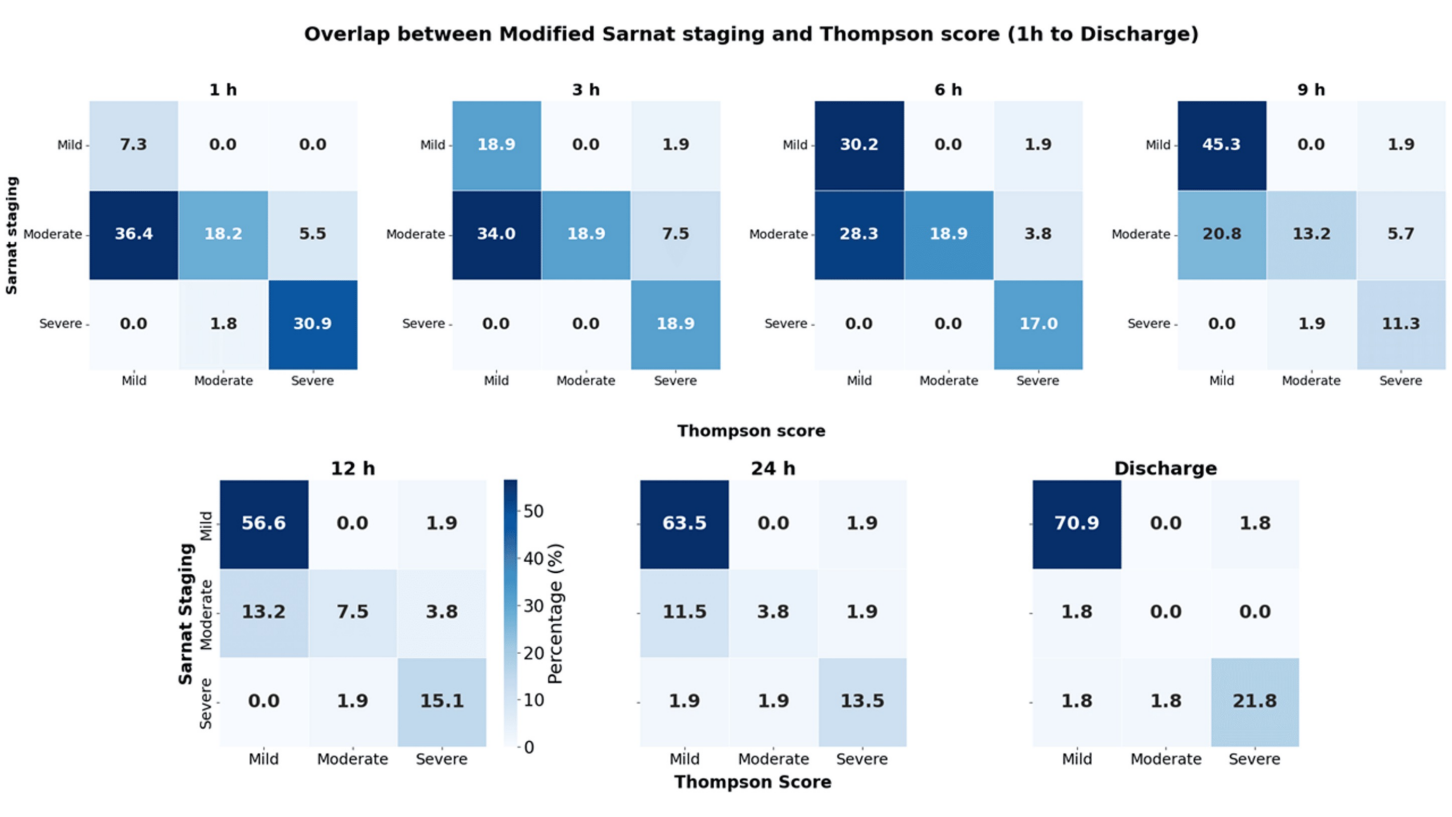
Heatmaps showing proportional overlap between Modified Sarnat Staging and Thompson Score severity categories from 1 hour of life through discharge.

As illustrated in Figure 1, heatmaps depicting proportional overlap between MSS and TS categories demonstrated distinct temporal patterns. In the early postnatal period (1-3 hours), the heatmaps were dominated by mild-moderate discordance, primarily attributable to a substantial proportion of infants classified as moderate encephalopathy by MSS but mild by TS. Despite this early discordance, severe classifications showed high concordance even at the earliest assessments, with no infants categorized as Thompson severe being staged as Sarnat mild.

During the mid-phase (6-12 hours), the heatmaps demonstrated progressive diagonal clustering, reflecting increasing alignment between MSS and TS categories. Over this interval, the proportion of discordant mild-moderate classifications decreased, while agreement within the moderate and severe categories increased.

By 24 hours of life, the heatmaps showed near-complete diagonal alignment, indicating maximal convergence between MSS and TS classifications. This convergence was further reinforced at discharge, with strong diagonal dominance across all severity categories, corresponding to clinically stable encephalopathy phenotypes and high agreement between the two scoring systems.

Time-efficiency

The mean time required to complete neurological assessments using the MSS and the TS at each assessment timepoint is summarized in Table 3. Across all evaluated timepoints from 1 to 24 hours of life, TS required consistently less time to complete than MSS.

**Table 3:** Comparison of assessment time required for Modified Sarnat Staging and Thompson scoring across serial timepoints.

Timepoint	Sarnat mean (s)	Thompson mean (s)	Mean difference (s)	P-value
1 hour	891.4	837.3	54.1	<10⁻⁹
3 hours	893.1	837.9	55.1	<10⁻⁹
6 hours	893.2	838.5	54.7	<10⁻⁹
9 hours	892.4	838.5	53.9	<10⁻⁹
12 hours	894.3	839.2	55.0	<10⁻⁹
24 hours	897.4	840.5	56.9	<10⁻⁹

The mean difference in assessment duration between the two scoring systems remained stable across timepoints, ranging from 53.9 to 56.9 seconds, with TS being faster at every assessment. At 1 hour of life, the mean assessment time was 891.4 seconds for MSS and 837.3 seconds for TS, and similar differences were observed at subsequent evaluations. The difference in assessment duration between MSS and TS was highly statistically significant at all timepoints (Wilcoxon signed-rank test, p < 10⁻⁹).

These findings demonstrate a reproducible and time-independent operational advantage of the TS over MSS during routine neurological assessment.

Progressive reduction in assessment time with examiner experience

**Figure 2:**
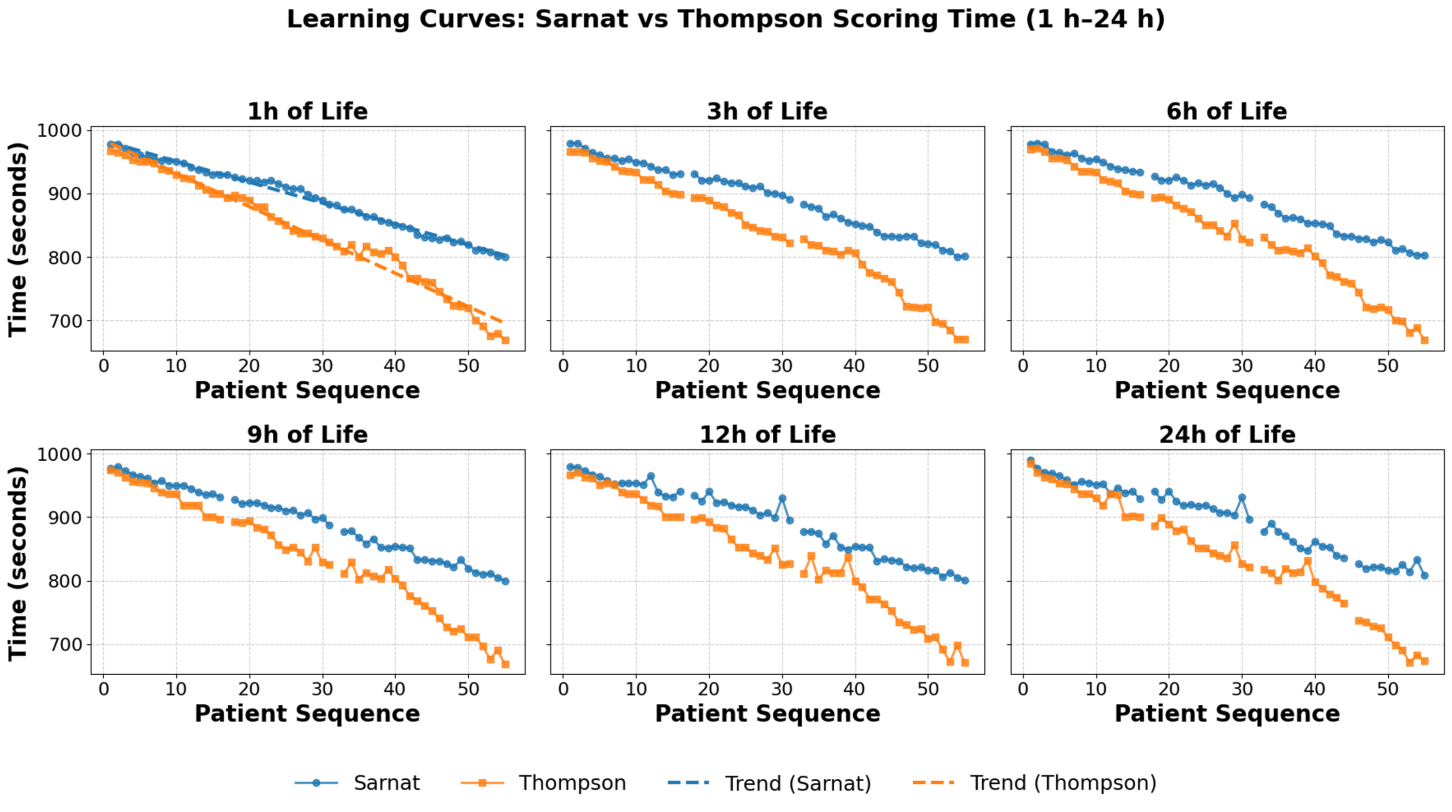
Progressive reduction in assessment time with increasing examiner experience for Modified Sarnat Staging and Thompson scoring across sequential patients.

Assessment time declined significantly across sequential patients for both scoring systems, as shown in Figure 2, demonstrating a progressive reduction in assessment duration with increasing examiner experience. Spearman’s correlation between patient sequence order and assessment time showed strong negative associations for both tools (ρ ≈ -0.98 for MSS and ρ ≈ -0.999 for TS), indicating substantial improvement in scoring efficiency with increasing clinical experience.

Despite progressive reductions in assessment time for both systems, the absolute time advantage of TS remained consistent throughout the study period, confirming that its greater efficiency was intrinsic to the scoring method and not solely attributable to examiner familiarity or progressive efficiency gains.

## Discussion

This study provides one of the most detailed comparative evaluations of the MSS system and the TS across multiple timepoints within the first 24 hours of life [9]. By combining serial clinical examinations, correlation analyses, weighted kappa agreement, cross-tabulation matrices, heatmaps, and time-efficiency metrics, this study offers a multidimensional understanding of how these two tools perform in real-world NICU conditions.

Correlation, agreement, and clinical interpretation of severity classification

The observed relationship between MSS and TS is consistent with prior studies demonstrating that early neurological assessment and electroencephalographic findings correlate with later neurodevelopmental outcomes in neonatal HIE [14,16,18]. Infants classified as having more severe encephalopathy by MSS staging consistently exhibited higher TS values, and this relationship became increasingly robust as the clinical picture evolved over time [16,18].

Importantly, agreement between the two scoring systems improved with advancing postnatal age. During the early postnatal period, neurological signs in HIE are often unstable and influenced by multiple transitional factors, including the effects of perinatal resuscitation, metabolic disturbances, evolving seizures, and cardiorespiratory adaptation [5,6]. As these confounding influences resolve and encephalopathy phenotypes become more clearly established, concordance between Sarnat and Thompson classifications strengthens. This explains the progressively better agreement observed at later timepoints and reinforces the importance of serial neurological assessments during the early postnatal period, particularly within the first 24 hours when clinical evolution is most dynamic.

Early discordance and borderline severity: clinical implications

A key observation from this study is the discordance between the two scoring systems during the early hours of life, particularly at the mild-moderate boundary. Within the first 1-6 hours after birth, a substantial proportion of neonates were classified as having moderate HIE by MSS but were categorized as mild by the TS. This discordance gradually diminished over time, with much closer agreement observed by 12-24 hours as neurological signs stabilized [8].

This early mismatch likely reflects fundamental differences in how the two systems capture neurological abnormalities. The MSS emphasizes global neurological depression, including alterations in consciousness, tone, posture, and primitive reflexes, features that may be subtle but clinically significant early in the disease course [12]. The TS assigns points across nine domains, including tone, level of consciousness, seizures, posture, Moro reflex, grasp reflex, suck, respiratory pattern, and fontanel tension, generating a composite score ranging from 0 to 22 [13]. Because the total score reflects cumulative abnormalities across these domains, early disease stages in which dysfunction is limited to one or two neurological systems may not immediately produce high aggregate scores. Perinatal metabolic derangements, including acidosis, have been shown to influence early neurological presentation and injury patterns in neonatal encephalopathy [19]. Consequently, in borderline cases, the TS may generate lower total scores despite clinically meaningful encephalopathy.

As a result, severity may be underestimated in some infants when relying solely on the TS during early assessments. These infants may initially appear to have mild encephalopathy, even though they are in the early stages of evolving moderate HIE that becomes more apparent with time and repeated examinations.

Impact of discordance on clinical decision-making

This early variability in classification has important practical implications for neonatal care. Several critical management decisions in HIE depend on the timely identification of moderate encephalopathy within a narrow therapeutic window [9,11]. Discordance between scoring systems at this stage can introduce diagnostic uncertainty and may influence the initiation, delay, or withholding of neuroprotective interventions.

An infant classified as mild by one system but moderate by another represents a clinical grey zone. In such situations, reliance on a single scoring tool may introduce uncertainty in severity staging. Current clinical practice emphasizes careful neurological reassessment during the early postnatal period, particularly within the therapeutic window for neuroprotective interventions. When discordance exists between mild and moderate categories, heightened clinical vigilance and serial neurological evaluation are warranted to avoid misclassification. Definitive management decisions should remain guided by comprehensive clinical assessment and established treatment criteria. Clinical seizures in neonatal encephalopathy are independently associated with adverse neurodevelopmental outcomes, further emphasizing the importance of accurate early severity classification [20].

Notably, severe encephalopathy showed high concordance between the two systems even during early assessments. No infant classified as severe by the TS was staged as mild by MSS, indicating reliable identification of severe disease by both tools. This consistency supports the robustness of both systems at the severe end of the spectrum, where clinical signs are more overt [12,13].

Time-efficiency, experience-related efficiency, and practical implications

An important operational finding of this study is the consistent time advantage of the TS over the MSS. Across all assessed timepoints, TS required approximately 55 seconds less per assessment, with the difference remaining highly statistically significant. While this time difference may appear modest for a single examination, its cumulative impact becomes meaningful in high-volume NICUs where repeated neurological assessments are performed within the first 24 hours of life. The faster completion time of the TS likely reflects its structured, itemized format, whereas Sarnat staging requires broader clinical synthesis across multiple neurological domains. The examiner had received prior training in both scoring systems before study initiation, and only active examination time was recorded; documentation time was excluded. The persistence of the time difference across sequential assessments, therefore, suggests that the observed efficiency advantage reflects structural differences between the scoring systems rather than progressive examiner learning alone.

Both scoring systems demonstrated progressive reduction in assessment duration with repeated use, reflecting increasing familiarity with structured neurological evaluation. This pattern is consistent with established principles of clinical skill acquisition, whereby structured scoring tools become more efficient with repetition. Importantly, the relative time difference between the two systems persisted throughout the study period, suggesting that the observed efficiency advantage relates to structural differences in scoring format rather than examiner learning alone. From a practical standpoint, in busy neonatal intensive care settings where repeated assessments are required within a limited therapeutic window, even modest per-examination time differences may accumulate and influence workflow efficiency. While both tools remain clinically valuable, the TS may be particularly suited for rapid serial monitoring, whereas the MSS system retains strength in detailed neurological characterization.

Temporal evolution and practical interpretation

The timing and evolution of brain injury play a critical role in the changing neurological phenotype observed in neonatal encephalopathy [21]. The progressive convergence of Modified Sarnat and Thompson classifications over time reflects the natural evolution of HIE. As encephalopathy stabilizes, neurological features become more distinct and interpretive variability decreases, resulting in near-complete agreement by 24 hours of life. From a practical standpoint, these findings support a complementary approach to neurological assessment. During the early postnatal period, particularly in borderline or evolving presentations, structural differences between the two scoring systems may influence severity categorization. The MSS system relies on integrated clinical interpretation across neurological domains and, in this cohort, tended to classify a greater proportion of infants as moderate in early assessments. In contrast, the TS assigns structured numerical values to individual domains, providing a simpler composite score and requiring less time to complete. In high-volume or resource-constrained neonatal intensive care settings where repeated monitoring is required, this relative simplicity and shorter assessment duration may offer operational advantages. However, because therapeutic decisions in HIE are closely linked to severity classification, comprehensive clinical evaluation remains paramount, particularly in cases where severity grading may influence intervention eligibility [22].

Strengths and limitations

The strengths of this study include its prospective, serial assessment design across multiple clinically relevant timepoints within the first 24 hours of life, allowing evaluation of the dynamic evolution of HIE. Use of a single trained pediatrician ensured internal consistency of assessments but precluded estimation of inter-rater reliability and may limit generalizability across examiners. This is the first study in which the correlation between MSS and TS has been extensively evaluated across multiple early timepoints. The use of both correlation and agreement statistics, complemented by cross-tabulations, heatmaps, and objective time-efficiency and experience-related efficiency analyses, provides a comprehensive and pragmatic comparison of the two scoring systems.

However, several limitations should be acknowledged. This was a single-center study conducted in a tertiary-care NICU, which may limit generalizability to other settings. The sample size, while adequate for correlation and agreement analyses, restricts detailed subgroup evaluation. Because missing data at later timepoints were considered missing not at random, agreement estimates at later stages may be influenced by survivor bias; however, early assessments, where discordance was greatest and clinical decisions are most time-sensitive, were largely complete. Therapeutic hypothermia was not available at the study center, which may affect the natural evolution of neurological findings and limit applicability to cooled populations.

## Conclusions

The MSS System and the TS demonstrate strong correlation and substantial agreement in the assessment of neonatal HIE, with concordance strengthening over time and with increasing severity of encephalopathy. In this cohort, the prevalence of HIE among neonates requiring NICU admission was 3.26% (227 of 6,965 deliveries), highlighting the significant institutional burden of this condition. The TS was consistently more time-efficient and exhibited marked experience-related efficiency advantages, supporting its utility in high-volume or resource-constrained neonatal intensive care settings. However, early discordance at the mild-moderate boundary underscores the importance of serial neurological assessments and the complementary use of both scoring systems during early clinical decision-making.
